# Altering the Ad5 Packaging Domain Affects the Maturation of the Ad Particles

**DOI:** 10.1371/journal.pone.0019564

**Published:** 2011-05-18

**Authors:** Raul Alba, Dan Cots, Philomena Ostapchuk, Assumpcio Bosch, Patrick Hearing, Miguel Chillon

**Affiliations:** 1 Center of Animal Biotechnology and Gene Therapy (CBATEG), and Department of Biochemistry and Molecular Biology, Universitat Autònoma de Barcelona, Bellaterra, Barcelona, Spain; 2 Department of Molecular Genetics and Microbiology, School of Medicine, Stony Brook University, Stony Brook, New York, United States of America; 3 Institut Català de Recerca i Estudis Avançats (ICREA), Barcelona, Spain; Queensland Institute of Medical Research, Australia

## Abstract

We have previously described a new family of mutant adenoviruses carrying different combinations of *att*B/*att*P sequences from bacteriophage PhiC31 flanking the Ad5 packaging domain. These novel helper viruses have a significantly delayed viral life cycle and a severe packaging impairment, regardless of the presence of PhiC31 recombinase. Their infectious viral titers are significantly lower (100–1000 fold) than those of control adenovirus at 36 hours post-infection, but allow for efficient packaging of helper-dependent adenovirus. In the present work, we have analyzed which steps of the adenovirus life cycle are altered in *att*B-helper adenoviruses and investigated whether these viruses can provide the necessary viral proteins *in trans*. The entry of *att*B-adenoviral genomes into the cell nucleus early at early timepoints post-infection was not impaired and viral protein expression levels were found to be similar to those of control adenovirus. However, electron microscopy and capsid protein composition analyses revealed that *att*B-adenoviruses remain at an intermediate state of maturation 36 hours post-infection in comparison to control adenovirus which were fully mature and infective at this time point. Therefore, an additional 20–24 hours were found to be required for the appearance of mature *att*B-adenovirus. Interestingly, *att*B-adenovirus assembly and infectivity was restored by inserting a second packaging signal close to the right-end ITR, thus discarding the possibility that the attB-adenovirus genome was retained in a nuclear compartment deleterious for virus assembly. The present study may have substantive implications for helper-dependent adenovirus technology since helper *att*B-adenovirus allows for preferential packaging of helper-dependent adenovirus genomes.

## Introduction

Adenovirus (Ad) is one of the most studied vectors in gene therapy and the most widely used vector in human clinical trials (http://www.wiley.co.uk/genmed/clinical). Helper-Dependent Ad (HD-Ad) alternatively referred to as Gutted, Gutless or High Capacity Ad, are promising vectors for gene delivery. The lack of any viral coding region in these vectors allows prolonged transgene expression due to a minimization of cellular immune responses [1], [2], [3]. In addition, they are able to incorporate genes up to 36 Kb in size. A helper virus is required in order to produce HD-Ad to provide all viral proteins *in trans*. Nevertheless, the production of infectious helper virus using this system must be inhibited. The use of recombinases to specifically excise the packaging domain (ψ) in the helper Ad genome represented an important advancement in HD-Ad production technology [4], [5]. At present, the most successful HD-Ad production system is based on excision of ψ from the helper virus genome mediated by Cre recombinase in combination with the physical separation of helper and HD-Ad virions by ultracentrifugation. Using this optimized system, levels of helper Ad contamination can, at best, be reduced to 0.1% 0.01% [6] (routinely about 1% -0.1%), the main limitation being the incomplete excision of ψ due to relatively low levels of Cre in adenovirus producing cells [7]. Of note, alternative packaging domain excision-based methods using recombinases such as FLPe has not resolved this issue [8], [9].

To address the limitations of the existing systems, we had previously reported a non-excision method based on the differential packaging efficiency between helper Ad and HD-Ad genomes mediated by the *att*B sequence of bacteriophage PhiC31 inserted between the inverted terminal repeat (ITR) and the packaging domain in the helper Ad genome [10]. The helper Ad packaging process is impaired 36 hours post-infection (hpi) rendering 100–1000 times lower *att*B-Ad titers due to a significant delay in the viral life cycle. This delay is extended up to 56–60 hpi. Interestingly, *att*B-FC31 technology is not dependent on the action of recombinases and, therefore, the generation of new recombinase-based cell lines can be avoided.

Ad has a strictly regulated infection cycle where multiple viral and cellular proteins interact to complete the viral replication program to generate infectious virus particles. *In vitro*, initiation of infection for Ad5 typically occurs when the fiber knob binds to the coxsackie and adenovirus receptor (CAR) in the cell membrane (or other receptors depending on the Ad5 protein [11] or the Ad serotype [12]), and penton base binds to alpha_v_beta_3_ or aλπηα_v_beta_5_ integrins leading to clathrin-mediated endocytosis [13]. After internalization, Ad5 escapes from the endosome following pH-acidification leading to viral capsid disassembly. Adenovirus particles traffick through the cell along the microtubule network [14] and reach the nucleus through nuclear pores [15]. Finally, adenovirus expresses the proteins required for viral genome replication and subsequent processes of the viral life cycle. Of note, genome replication and capsid assembling events occur in different nuclear compartments [16].

Packaging of the adenoviral genome is a complex process where different viral proteins, (e.g. L1-52/55K, IVa2 and L4-22K), interact with ψ to encapsidate the viral genome in a polar process to form a mature viral particle [17]. Interestingly, the packaging domain can be located at either end of the viral genome allowing for viral genome packaging. The distance of ψ from the 5′ or 3′ ends of the genome is crucial for optimal packaging activity, although some flexibility in its location is tolerated. For example, when the distance between the ITR and ψ is 655 nucleotides or more, virus viability is severely compromised. However, when the distance between the ITR and ψ is increased up to 271 nucleotides, fully viable viruses are produced [18]. During the Ad assembly process, the density of the viral particle varies from 1.29 to 1.35 g/cc which appears to reflect the insertion of viral DNA into an empty capsid, the subsequent packaging process, followed by final virion maturation [17]. The Ad protease, also called adenain, is transported into the capsid and mediates the final steps of mature particle formation via the cleavage of a number of virion proteins including pIIIa, pVI, pVII, pVIII, pTP, X and L1-52/55K [19].

For final application of *att*B-helper adenoviruses in HD-Ad production, an extensive characterization of these vectors is required. Here, we have investigated which processes of the attB-helper Ad viral life cycle are affected.

## Methods

### Adenovirus generation, production and purification

Ad5/*att*P, Ad5/RFP, Ad5/βgal and *att*B-helper Ads (Ad5/FC31.1 or Ad5/FC31.2) were produced at the Vector Production Unit in the Center of Animal Biotechnology and Gene Therapy at the Universitat Autònoma de Barcelona (Bellaterra, Spain) as previously described [10]. Briefly, *Pac*I-linearized plasmids were transfected into HEK293 cells (ATCC, CRL-1573) and virus recovered 8–10 days post-transfection. Then, viruses were amplified through successive 56–60 hour infection cycles until a total of 4×10^8^ HEK293 cells were infected. Ad5/FC31.1[ψ] was generated using Stow's method [20], by cotransfecting *Pac*I-linearized pAd5/FC31.1 plasmid and in340 virus complete genome (with the exception of the left terminus). Plaques were isolated and different clones were amplified to generate Ad5/FC31.1[ψ] as previously described. Viral mutant Ad5/ts369 was produced in HEK293 cells grown at 32.5°C. Viruses were purified using two consecutive CsCl gradients (a step gradient followed by an equilibrium gradient [21]) followed by elution on a Sephadex PD-10 desalting column (Amersham Biosciences, Uppsala, Sweden). For *attB*-helper Ads, both, mature and immature particles from the first gradient were purified in the second cesium chloride gradient. Final purified viral stocks were titered by determining their concentration (particles/ml) by optical density at 260 nm (1 OD260 unit = 1×10^12^ particles/ml), and their infectivity (infectious units/ml) was measured by endpoint dilution assay. Briefly, end-point dilution assays were performed in triplicate by infecting 293 cells with serially diluted vectors, and then counting the number of transgene (GFP, RFP or βgal) expressing cells. Viral titers ranged between 0.7×10^10^ to 3×10^10^ IU/ml with an average ratio of physical particles to infectious units of 40∶1.

### Viral production assay

HEK-293 cells were infected with control Ad and *att*B-helper Ad at 5 IU/cell in a 6-well format. Fresh DMEM medium was added 6 hpi. Pellet and supernatant were harvested at 36 and 56 hpi and three rounds of freeze/thawing performed to liberate viral particles. Finally, infectious titer was calculated at each time point by end-point dilution assay in three independent analysis.

### Southern Blot analysis of viral genomes

High molecular weight DNA was extracted following Hirt's Method [22]. For packaged genomes, DNA was extracted from 10 µl of purified virus or from 200 µl of crude viral lysate. Fourteen µl of 10% SDS, 6 µl of 0.5 M EDTA and 40 µl of 20 mg/mL proteinase K (Roche,) were added. Samples were incubated for 3 hours at 55°C and later heated for 5 min at 95°C to liberate viral genomes. Samples were then diluted to 200 µl with deionized water and 100 µl of 7.5 M ammonium acetate added. Viral DNA was extracted using 300 µl of Phenol/CHCl_3_/isoamyl alcohol (25∶24∶1) and absolute ethanol, then precipitated by washing twice with ethanol 70%. Pellets were then dissolved in 50 µl of sterile deionized water. *Spe*I-digested viral genomes were separated by gel electrophoresis, and then incubated in: a) 200 mM HCl (15 min); b) 5 N NaOH, 1.5 M NaCl (45 min); c) 20× SSC buffer (NaCl 3 M, Sodium Citrate 0.3 M pH = 7.4) (45 min). DNA was transferred to a positively-charged membrane (Roche Diagnostics Corp, Indianapolis) in 10× SSC buffer for 18 hours, fixed via exposure to UV light with a UV-stratalinker 1800 (Stratagene, La Jolla, CA) and detected using Alkphos Direct Labelling kit (Amersham Biosciesces) according to manufacturer's instructions.

### Viral replication assay

Both Ad5/*att*P and Ad5/FC31.1 were used to infect HEK-293 cells at 5 IU/cell in a 6 well plate for 6 hours, in two independent experiments. Cell were washed and fresh medium added. Cell pellets/supernatants were recovered at 24, 28, 32 and 36 hpi. Viral DNA was extracted from one tenth of the crude lysate and transferred to a Hybond-XL membrane (Amershan Biosciences) and detected using a GFP probe (at concentrations ranging from 50 to 0.01 ng). The GFP probe was obtained by isolating a 1597 bp product corresponding to the GFP expression cassette of pKS/RSV (kindly provided by Eric Kremer, Montpellier, France) using a *Spe*I+*Sal*I restriction digest. The product was then labelled with AlkPhos Direct Labeling Kit and used to probe viral DNA using the DP-Star detection kit according to the manufacturer's instructions. In parallel, crude lysates from infected cells were analyzed in triplicate by end-point dilution assay to calculate the number of IU/cell at each time point.

### Co-infection assay

HEK-293 cells were infected with Ad5/*att*P, Ad5/FC31.1 and Ad5/FC31.2 (all carrying a GFP cassette) or Ad5/βgal Ad (carrying a βgal cassette) in a 6-well plate at 5 IU/cell for single infection. For co-infection experiments *att*B*/att*P-modified Ads (Ad5/*att*P, Ad5/FC31.1 and Ad5/FC31.2) were co-infected with control Ad5/βgal at 5 IU/cell per virus. At 36 hpi, viruses were harvested and further titered by end-point dilution assay using fluorescence microscope and X-gal staining for GFP or βγαλ ϖιρυσεσ ρespectively.

### Flow Cytometry

4×10^6^ HEK293 cells were infected (n = 5) with different control and helper Ad (MOI = 2). Infected cells were recovered at 12, 24, 30 and 36 hpi. Medium was recovered and saline buffer was added to harvest the cells. After centrifugation, cells were first resuspended in PBS buffer (1×) and then in 4% paraformaldehyde buffer. Finally, GFP expression by infected cells was analyzed by flow cytometry at *Servei de Citometria* of IBB-UAB (Universitat Autònoma de Barcelona).

### Electron Microscopy assay

Viral particles were analyzed by the Uranyl Acetate method as previously described [23]. The samples were viewed with a FEI Tecnai 12 BioTwinG2 transmission electron microscope at 80 kV and the digital images were obtained with an AMT XR-60 CCD Digital Camera System. Services were provided by the TEM Facility, Central Microscopy Imaging Center at Stony Brook University, Stony Brook, New York, USA.

### Silver Stain SDS-PAGE protein analysis

Viral proteins from virions purified by CsCl equilibrium gradient centrifugation were separated by electrophoresis on a SDS-10% polyacrylamide gel and silver stained as previously described [24]. Gels of viral proteins were incubated with 50% methanol/10% acetic acid followed by 10% methanol/5% acetic acid incubation, dithiothreitol, and 12 mM silver nitrate. Signal was developed by incubation in 2% potassium carbonate containing 0.044% formaldehyde, and development stopped by incubation in 1% acetic acid. Finally, the gel was washed with distilled water.

### Western Blot and immunodetection analysis

Viral proteins were separated by SDS-polyacrylamide gel electrophoresis and transferred to a Hybond-P membrane (Amersham-Pharmacia) by standard methods, in at least two independent experiments. Membranes were probed with antibodies directed against penton base and hexon (generous gifts of Carl Anderson, Brookhaven National laboratory, USA), L1-52/55K [25], pVII/VII [26] and β-actin (ref. a2066, Sigma). Proteins were visualized using AlkPhos-coupled secondary antibody (Zymed) and a fluorescent substrate (AttoPhos Substrate, Promega). Signals were analyzed using a phosphorimager (Molecular Dynamics Storm 860).

### EMSA assay

Nuclear extracts prepared from HEK293 and DKzeo cells [27] were in HEPES 20 mM at pH 7.5, glycerol 20%, NaCl 450 mM MgCl_2_ 1.5 mM, EDTA 0.2 mM in sterile deionized water and protease inhibitors, following the protocol described by Zhang et al [28], and stored at −80°C. Protein extracts were quantified using BCA following the manufacturer's protocol (Pierce). Three µg of nuclear extract were incubated for 30 minutes at 37°C with 2.6 µl 5× Binding buffer (LightShift Chemiluminescent EMSA Kit, 20148X, Pierce), 1 µl poly-deoxyinosic-deoxycytidylic, and 1 µl of biotin-labeled *wild type-att*B or *mutant att*B. The samples were then separated in a non-denaturing polyacrylamide gel for 90 minutes at 120 V, and transferred to a positively charged membrane (Roche Diagnostics Corp, Indianapolis,). Subsequently the membrane was incubated with a peroxidase-streptavidin conjugate, followed by washing and incubation with luminol (PIERCE) following the protocol provided by the manufacturer. To confirm observations, the experiment was repeated in three independent analysis. *att*B*wt* sequence (*wild type*): 5′ACCGGTCCGCGGTGCGGGTGC CAGGGCGTGCCCTTGGGCTCCCCGGGCGCGTACTCCAC3′. attB* sequence (mutant): 5′ACCGGTGGGCACGCGCGCACCTGGCGCACCGCGTCGGCGCACCTGCGCACCTG GCACCA3′.

### Statistical Analysis

Statistical calculations were performed using the G-Stat version 2.0 statistical program. Statistical significance was determined by one way ANOVA test with P value set at ≤0.05. Data are presented as mean ± SD unless stated otherwise.

## Results

### attB/attP sequences flanking the packaging signal do not affect the entry of the adenoviral genome into the nucleus

**Figure 1:**
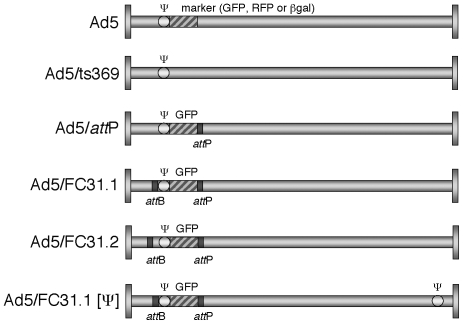
Adenovirus constructs used in this work containing the packaging signal flanked by different combinations of *att*B/*att*P sequences and a reporter expression cassette. Viral genomes schemes are not to scale.

**Figure 2:**
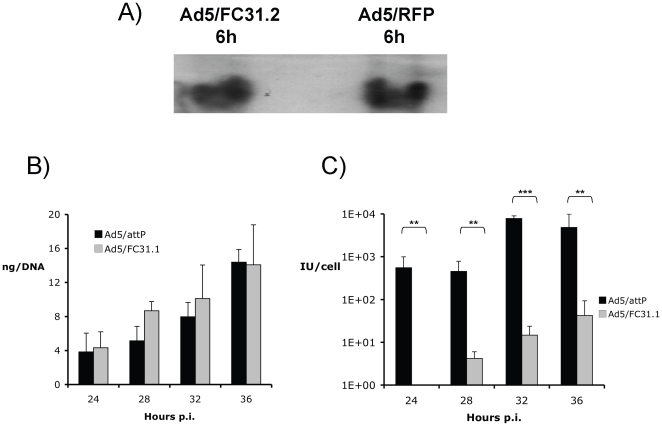
Southern Blot analysis of Ad5/RFP and *att*B-genome at 6 hpi in 1×10^7^ HEK293 cells (MOI = 5) (A) Equal amounts (30 µg) of high molecular weight DNA were loaded. Probe used contains the first 194 nt of adenovirus genome. (B) Viral genome replication of Ad5/*att*P and *att*B-Ad. Viral DNA produced at 24, 28, 32 and 36 hpi in HEK-293 cells (MOI = 5) were quantified by Dot-Blot in triplicate and titers measured in nanograms (ng) of DNA. (C) Virus yield of Ad5/*att*P and *att*B-Ad. Infectivities of vectors were analyzed in triplicate, and further quantified in IU/cell (in bars) by fluorescent microscopy after a secondary infection in HEK-293 cells. Statistics were performed with natural log values using one way ANOVA (** p<0,01; *** p<0,001).

The recombinant viruses used in this study include viruses with RFP (Ad5/RFP) or GFP (Ad5/*att*P, Ad5/FC31.1, Ad5/FC31.2) expression cassettes in place of the E1 region. Ad5/*att*P contains a PhiC31 *att*P site located to the right of the GFP gene. Ad5/FC31.1 and Ad/FC31.2 contain the same *att*P insertion as well as an additional PhiC31 *att*B site inserted to the left of the Ad5 packaging domain and differ only by the additional insertion of a 65-bp spacer between *att*B and ψ in Ad5/FC31.2 (Fig. 1). We previously reported that the *att*B insertion, but not the *att*P insertion, reduced viral DNA packaging, delayed the production of infectious virus and decreased overall infectious virus yield [10]. Here, we characterize this defect in further detail. Entry of *att*B-Ad genomes (adenovirus vectors carrying the attB sequence 5′ of the packaging signal) into the nucleus was compared to control first generation Ad5/RFP by infecting HEK293 cells at a multiplicity of infection (MOI) of 5 infectious units per cell (Figure 2A). Viral genomes were isolated by Hirt's method from the nucleus at 6 hpi. Southern blot densitometry showed that both viral genomes of control and *att*B-Ad vectors reached the nucleus at similar levels, indicating that viral proteins involved in virion trafficking must be present and fully active in *att*B-Ad.

### Viral DNA replication and protein production of attB-adenovirus are not affected

**Figure 3:**
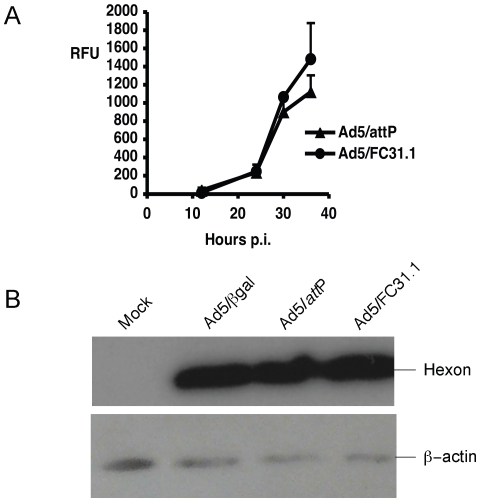
Analysis of protein expression. HEK-293 cells were infected with Ad5/βgal, Ad5/*attP* or *att*B-Ad at MOI of 5, and samples were analyzed at 24, 30 and 36 hpi. Non-viral marker GFP protein was quantified by measuring relative mean fluorescence intensity per cell (RFU/cell) by Flow Cytometry (A). Late viral hexon protein was detected from protein extracts by Western Blot analysis at 24 hpi (B). Equal amount of proteins were loaded and β-actin protein was used as control. Mock are uninfected HEK-293 cells.

To analyze whether viral DNA replication was altered, viral genome replication was quantified at 24, 28, 32 and 36 hours post infection (hpi) and correlated with infectious virus yields at the same time points. HEK-293 cells were infected with *att*B-Ad and Ad5/*att*P (control Ad) at a MOI of 5, and viral DNAs purified and quantified by dot-blot analysis. We found that for both *att*B-Ads, the kinetics and level of genome replication were similar to Ad5/*att*P at all time points (Ad5/FC31.1, Figures 2B and 2C; Ad5/FC31.2, data not shown). However, the amount of infectious *att*B-Ad (in IU/cell) was markedly reduced (>99%) at all times compared to control Ad, and statistically significant (p<0.01 at 24, 28 and 36 hpi; and p<0.001 at 32 hpi). These results showed that the delayed life cycle of *att*B-Ads must be due to alterations in subsequent steps to replication. Moreover, since Ad5/FC31.1 and Ad5/FC31.2 vectors are identical (except for an extra 65-bp spacer between *att*B and ψ in Ad5/FC31.2) and have a similarly delayed viral life cycle (Reference 8, Figure 3), as well as similar viral infectivity and DNA replication, they were used interchangeably throughout the experiments.

In addition to viral replication, the levels of expression of several viral proteins from the Ad5/βgal, Ad5/*att*P or *att*B-Ad genomes were also analyzed. Both the GFP-marker protein (driven by a constitutive promoter and analyzed at 24, 30 and 36 hpi; Figure 3A) and Ad5 hexon protein (driven by the endogenous adenoviral MLP promoter and analyzed at 24 hpi; Figure 3B) were expressed at similar levels from the *att*B-Ads compared to the control Ads. These results support our notion that the delayed maturation of *att*B-Ad is due to effects on life cycle steps following viral genome replication.

### Adenovirus proteins provided *in trans* do not normalize the viral life cycle of attB-Ad vectors

**Figure 4:**
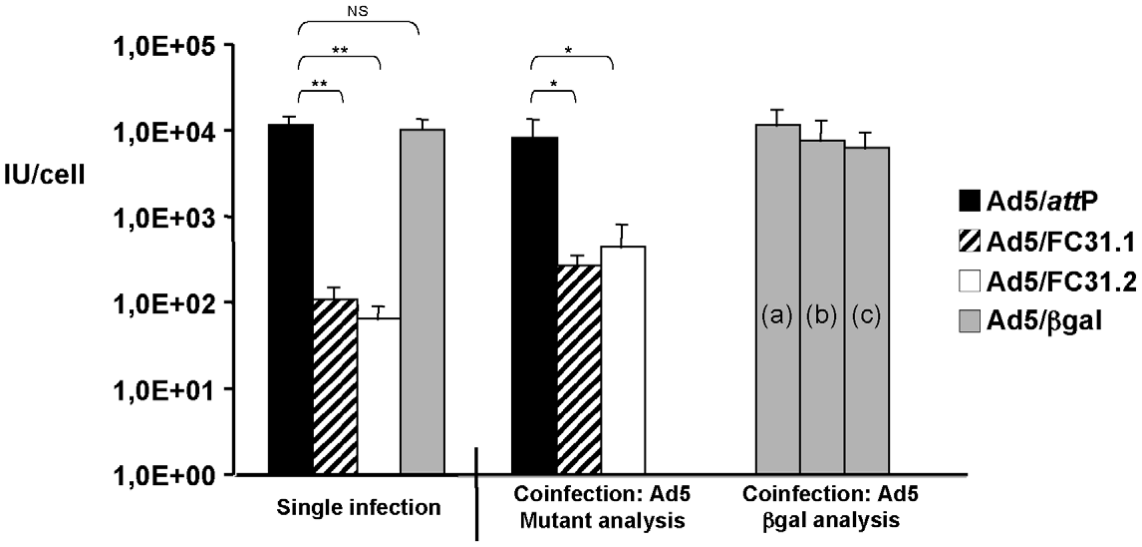
Co-infection experiments using Ad5/βgal and *att*B/*att*P vectors (Ad5/*attP* and *att*B-genomes, all expressing GFP). HEK-293 cells were infected at 5 IU/cell for each adenovirus vector (“single infection”), or co-infected with Ad5/βgal and different *att*B/*att*P-containing vectors (5 IU/cell each vector). (a) Refers to Ad5/βgal coinfected with Ad5/*attP*; *(b) to* Ad5/βgal coinfected with Ad5/FC31.1; and (c) to Ad5/βgal coinfected with Ad5/FC31.2. At 36 hpi, virus were harvested and further titered by end-point dilution and analyzed in triplicate by βgal or GFP expression in two independent experiments. Asterisks refers to statistical significance: * p<0,05; ** p<0,01.

Previous results showed that *att*B-Ads could rescue HD-Ad vectors [10] and are in agreement with results here suggesting that the defect in attB-Ads is a *cis*-acting effect. We examined this issue by testing whether the delay in the accumulation of infectious *att*B-Ad could be rescued by co-infection with a control adenovirus. *att*B-Ads and Ad5/*att*P (carrying a GFP cassette) were co-infected with a first generation adenovirus (Ad5/βgal) and analyzed at 36 hpi. As expected, single infections of control Ad5/*att*P or control Ad5/βgal (MOI of 5) generated titers 100–200-fold higher than those from *att*B-Ad vectors (Figure 4). Similarly, in co-infection experiments using Ad5/βgal (MOI of 5) either with *att*B-Ad (MOI of 5) or with control Ad5/*att*P (MOI of 5), only modest changes in viral titers (either of Ad5/βgal, Ad5/*att*P or *att*B-Ad) compared to the single virus infection were observed. These data show that the delay in accumulation of infectious *attB*-Ad cannot be rescued (or rescued only in a minor fraction) to levels of control Ad5/attP (p<0.05 for both attB-Ad vectors) by the presence of viral proteins provided *in trans* by an Ad vector with a normal life cycle.

### Altered viral capsid formation of attB-Ad vectors at 36 hpi

**Figure 5:**
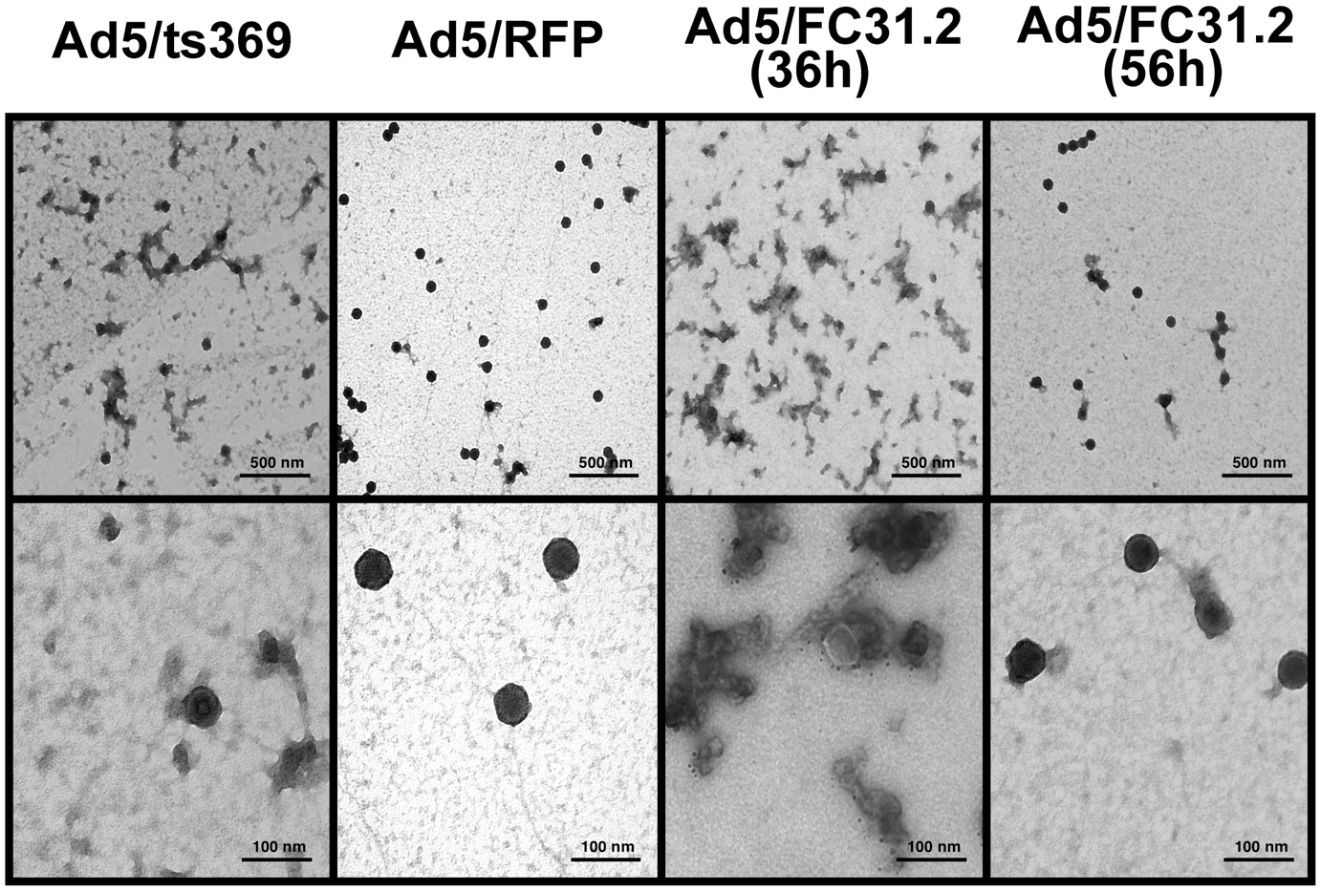
Electron microscopy analysis of CsCl-purified Ad5/ts369 and Ad5/RFP viruses at 36 hpi and *att*B-Ad at 36 and 56 hpi in HEK293 cells. Uranyl acetate staining was used to visualize viral particles. Representative sections among multiples pictures are shown. Bar equals 500 nm on top row and 100 nm in bottom row.

Since the delay in the accumulation of mature *att*B-Ad was independent of replication and expression of the structural proteins, we examined the integrity and formation of adenoviral capsids by electron microscopy (Figure 5). As controls, Ad5/RFP and Ad5/ts369 were analyzed at 36 hpi and *att*B-virus was analyzed at 36 and 56 hpi. Ad5/ts369 is an L1-52/55K temperature-sensitive mutant virus that is blocked at an intermediate stage when grown at the non-permissive temperature of 39.5°C [29]. It accumulates light intermediate particles and was used as a control for intermediate virus assembly. Interestingly, at 36 hpi *att*B-Ad appeared to be in an intermediate state of assembly which resembled Ad5/ts369 grown at the non-permissive temperature. Protein aggregates were observed along with relatively few virus-like particles. The particles appeared to lack DNA since they accumulated uranyl acetate in the interiors. However, at 56 hpi, most *att*B-Ad capsids appeared mature and resembled those observed for control Ad5/RFP at 36 hpi (Figure 5).

### Maturation of attB-Ad particles is severely impaired at 36 hpi

**Figure 6:**
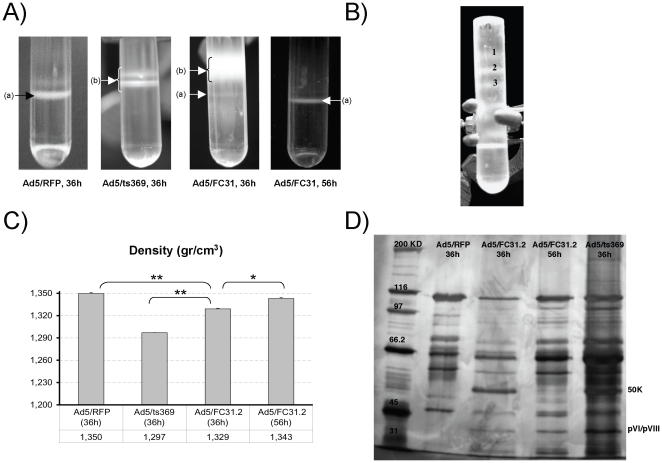
Results of first cesium chloride gradient (A). Shown are images for Ad5/RFP and Ad5/FC31.2 at 56 hpi and Ad5/FC31.2 at 36 hpi. Arrows indicate (a) mature particles, and (b) immature particles. (B) Second cesium chloride gradient for Ad5/FC31.2 at 56 hpi, 1, 2 and 3 indicates three low density bands. (C) Density in (g/cm^3^) of purified particles of Ad5/RFP, Ad5/ts369, Ad5/FC31.2 at 36 hpi and Ad5/FC31.2 at 56 hpi. (D) Silver stained polyacrylamide gel of CsCl purified particles of Ad5/ts369 and Ad5/RFP at 36 hpi. and *att*B-Ad at 36 and 56 hpi. Asterisks refers to statistical significance: * p<0,05; ** p<0,01.

Inefficient virus maturation was also evident during the purification process on cesium choride gradients. Ad5/FC31.2 (36 hpi) produced mainly immature particles compared to control Ad5/RFP (36 hpi) or Ad5/FC31.2 at 56 hpi (Figure 6A). Results of a second isopycnic cesium chloride gradient centrifugation of Ad5/FC31.2 (56 hpi) revealed the presence of at least three immature intermediates of maturation, termed Band 1, Band 2 and Band 3, (Figure 6B) which were individually isolated for further characterization. Of note, density of the purified Ad5/FC31.2 (36 hpi) particles was significantly lower with respect to Ad5/RFP (36 hpi) and Ad5/FC31.2 (56 hpi) particles densities (Figure 6C).

The virus maturation process was analyzed in detail by silver staining of viral proteins of purified particles of Ad5/RFP (36 hpi), Ad5/ts369 (36 hpi) grown at the nonpermissive temperature and *att*B-Ad (36 and 56 hpi) (Figure 6D). The protein banding pattern of *att*B-Ad at 36 hpi was similar to that of the Ad5/ts369 light intermediate particle protein pattern. Both showed the presence of precursor proteins including pVI, pVIII and 50K. In contrast, at 56 hpi *att*B-Ad presented a similar protein pattern to Ad5/RFP (e.g., the loss of the 50K protein). However, not all *att*B-Ad particles were fully mature since pVI and pVIII precursors were still detected.

**Figure 7:**
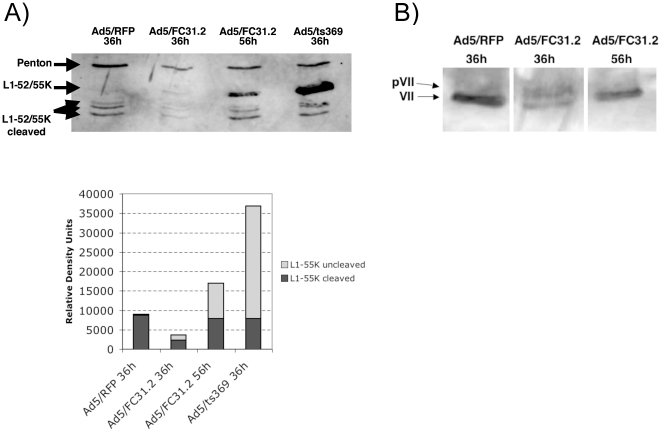
Analysis of L1-52/55K and pVII proteins of *att*B-adenovirus. (A) Western Blot analysis of penton base (control for protein content) and L1-52/55K viral proteins from CsCl gradients of Ad5/RFP and Ad5/ts369 at 36 hpi and *att*B-Ad at 36 and 56 hpi (cleaved and uncleaved L1-52/55K protein are indicated by arrows). Densitometric analysis of the Western-blot for the L1-52/55K cleaved and uncleaved proteins after normalization to penton base. (B) Western blot analysis of adenoviral protein pVII from CsCl gradients of Ad5/RFP at 36 hpi and *att*B-Ad at 36 and 56 hpi.

L1-52/55K has been shown to be cleaved during the assembly process; likely by adenain [16]. Intermediate and empty particles contain intact L1-52/55K as well as cleaved products of approximately 40 KDa, while the mature virus lacks intact L1-52/55K and is markedly reduced for the 40K product. Western blot analysis for L1 52/55K of Ad/RFP, Adt5/ts369 and *att*B-Ad5 indicated that at 36 hpi, particles of Ad/RFP did not contain intact L1-52/55K (although the 40K cleavage products were observed); while light intermediate particles of Ad5/ts369 grown at the non-permissive temperature contained both the intact and cleaved product(s) of L1-52/55K (Figure 7A). However, at 36 hpi *att*B-Ad produced very low levels of L1-52/55K, suggesting that only few capsids had incorporated this protein. At 56 hpi, the level of cleaved L1-52/55K proteins increased, though a significant percentage remained uncleaved (54%) indicating that part of the *att*B-Ad viral population was still at an intermediate step of maturation. Analysis of pVII showed that at 36 hpi *att*B-particles had similar levels of precursor and mature protein VII (60% to 40%, respectively), while at 56 hpi protein VII was mature (Figure 7B). As expected, protein VII was unprocessed in the immature particles isolated from Band 2 and 3 (Figure 6B) and not present in Band 1, indicating that these putative intermediates corresponded to capsids at the very early steps of maturation (Figure S1). In addition, material from these bands did not contain viral DNA and were not infectious.

### A second packaging signal cloned at 3′-ITR normalizes infectivity levels of attB-Ad

**Figure 8:**
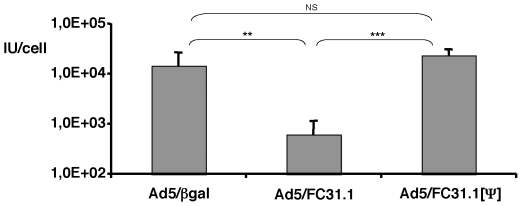
Effect of a second packaging signal in *att*B-Ad production. Viral production in IU/cell of Ad5/FC31.1, Ad5/FC31.1[ψ] and Ad5/βgal at 36 hpi. in HEK293 cells. Titering was performed in triplicate in HEK-293 cells. Asterisks refers to statistical significance: ** p<0,01; *** p<0,001; NS = not significant).

To determine whether the defect in virus assembly observed with *att*B-Ad was due to a dominant effect mediated by the *att*B sequence we generated a new Ad construct named Ad5/FC31.1[ψ] with a second packaging domain inserted at the right-end of the genome (Figure 1). Results of infections showed that at 36 hpi similar levels of infectious viral particles of Ad5/FC31.1[ψ] as compared to the control Ad (Ad5/βgal) were obtained (Figure 8) indicating that Ad5/FC31.1[ψ] capsids were fully matured at that time. This result show Ad5/FC31.1[ψ] viral genomes allow for normal genome replication and viral protein expression at 36 hpi, suggesting that the delayed *att*B-helper genome life cycle is not due to irreversible trapping in a nuclear compartment that inhibits virus assembly.

### A nuclear protein interacts with the *att*B sequence in vitro

**Figure 9:**
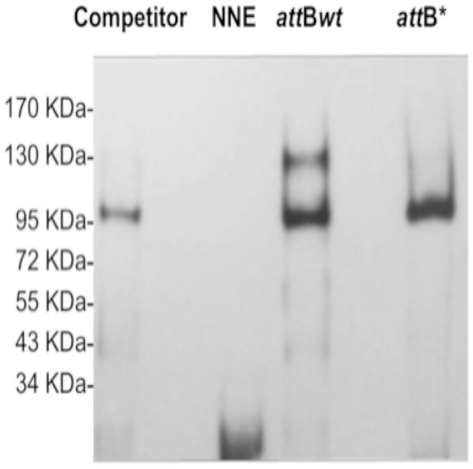
Enzyme electrophoretic mobility shift assay using HEK293 cell nuclear extracts. Competitor: HEK293 cell nuclear extracts incubated with the *attBwt* sequence together with poly-dIdC and unlabeled *attBwt* as specific competitor. NNE: *att*B*wt* sequence without nuclear extracts. *att*B*wt*: Nuclear extracts from HEK293 cells incubated with a wild type *att*B sequence and poly-dIdC. *att*B*: Nuclear extracts from HEK293 cells incubated with a mutant *att*B sequence and poly-dIdC.

Taken together the results, with Ad-helper vectors with the *att*B sequence 5′ of the packaging signal having a delayed viral cycle leads to the hypothesis that a cellular factor could be interacting with the *att*B sequence, impairing the formation of the packaging complex with ψ. In order to test this hypothesis an electrophoretic mobility shift assay (EMSA) was performed to detect specific interactions mediated by nuclear protein(s). In addition to the *wild type att*B sequence, we also tested a mutant *att*B sequence encoding the same nucleotide composition as the *wild type att*B but in a different order. Results clearly show that *in vitro*, the *wild type att*B sequence (but not the mutant *att*B) interacts with a nuclear protein from HEK293 cells (Figure 9). In order to test whether this interaction was specific for the HEK293 cell line, we also tested the interaction between the *att*B sequence and protein extracts from canine DKZeo cells. The band-shift also observed in DKzeo cells (data not shown), indicating that this interaction was not specific for HEK293 cells. The band-shift in HEK-293 cells appeared to be more intense than in DKzeo cells, suggesting different levels of expression for this nuclear factor between both cell types.

## Discussion

Helper-dependent adenovirus vectors are important candidates for gene therapy due to their reduced capacity to induce cellular immune responses and their ability to direct stable transgene expression of up to 2 years [30]. Despite these advantages, their production presents two important problems: (1) contamination with helper Ad and (2) low titer preparations. During the last decade, optimization of the Cre-*lox*P system has been done by combining the excision of ψ (using a Cre expressing cell line) with the physical separation of helper and HD-Ad virions by density in CsCl ultracentrifugation. This has improved the reduction of helper Ad contamination levels from 10% down to 0.1%-0,01% [6]. Additional strategies for optimizin the excision of ψ mediated by Cre recombinase must consider the compromise required between recombination activity and cell toxicity, i.e. low Cre levels limit efficient excision of ψ, while high Cre levels become cytotoxic and affect proliferation of adenovirus producing Cre-cell lines [7]. Moreover, the need for large amounts of HD-Ad vectors for clinical assays in human patients prevents the use of non-scalable downstream processes such as ultracentrifugation. Therefore, to improve production and purification of HD vectors beyond the limits of current systems, other strategies must be developed.

We have recently generated a new system based on preferential packaging of the HD-Ad genome compared to the helper Ad genome. We constructed a family of helper Adenovirus by flanking the packaging signal with PhiC31 *att*B/*att*P sites [10]. At 36 hpi production yield of *att*B/*att*P Ads (in IU/cell) is significantly reduced (>99%). Surprisingly, though *att*B and *att*P sites are sequences recognized by FC31 recombinase, differential packaging takes place in the absence of recombinase indicating that mechanism(s) other than recombination are involved.

Here, we have analyzed the mechanism underlying the delayed viral cycle of *att*B-Ad by studying the steps in adenovirus particle generation. We have observed that *att*B-Ad can reach the cell nucleus at the same time (6 hpi) and at the same level (genome copies) as a control Ad (Ad5/RFP). Since at this time replication has not yet started, thus the observed viral genomes are derived from the incoming virus. This indicates that *att*B-Ad viral capsids allow appropiate cell trafficking and uncoating, and therefore viral capsid proteins (like adenain and pVII) must be fully active. When compared to control Ad5, viral DNA replication from *att*B-Ad and *att*P-Ad vectors is slighty reduced (between 0% and 50%, data not shown). However, this does not affect the kinetics and level of virus production since Ad5/*att*P behaves exactly as control Ad5. Moreover, quantification of viral DNA replication up to 36 hpi showed that although *att*B-Ad infectious titers were 2 to 3 logs lower than those for control *att*P-Ad, attB-Ad replicated to the same level as *att*P-Ad at all time points. Therefore, viral genome replication is not causing the delay in the *att*B-Ad viral life cycle.

Analysis of adenoviral gene expression showed that protein expression levels from *att*B-Ad were equivalent to controls (Ad5/*att*P or Ad5/βgal). Consistent with this result, coinfection of *att*B-adenoviral particles with Ad5/βgal, which does not show any delay in viral life cycle, did not rescue the delay in the *att*B-Ad5 life cycle. Furthermore, as previously shown, *att*B-Ad viruses function efficiently as helper virus for the production of HD-Ad vectors [10]. Therefore, the cause of the delayed viral cycle must reside in the genome sequence or the viral genome structure of *att*B-Ad.

The delay in packaging and accumulation of infectious *att*B-Ad genomes was rescued by the incorporation of a packaging domain at the right end of the *att*B-Ad genome. Therefore, the *cis*-acting defect is recessive to the presence of a wild type packaging domain. This result indicates first, that the *att*B-helper genome is not irreversibly retained in a nuclear compartment inhibitory to virus assembly, and second, that packaging impairment was not caused by spontaneous and deleterious mutations in the *att*B-Ad genomes.

We have previously reported that less than 5% of the *att*B-Ad genomes are packaged at 36 hpi in coinfection experiments compared to control viral genomes [10]. Furthermore, only a minimal fraction of the packaged *att*B-genomes were able to form infectious particles at 36 hpi [10]. To test if differences in *att*B-Ad infectivity correlated with differences in capsid morphology, adenoviral capsids were analyzed by electron microscopy. As observed for the control Ad5/βgal at 36 hpi, the majority of *att*B-Ad vectors consisted of full capsids at 56 hpi. In contrast, at 36 hpi, *att*B-particles were present in an immature state consisting of protein aggregates and some empty particles; similar to that observed for the light assembly intermediate mutant Ad5/ts369 grown at the nonpermissive temperature. Since viral proteins provided by *att*B-Ad allow normal packaging of HD-Ad at 36 hpi [10] then, according to the classical theory of adenovirus assembly [17], pre-formed empty capsids should be also available for the packaging of *att*B-Ad genomes at this time. However, instead of empty capsids, electron microscopy images showed an accumulation of unstructured protein aggregates with few empty capsids. These results are suggestive of a modified theory; that is, that the packaging complex on the viral DNA acts as an initiator of the formation of a procapsid/DNA assemblage followed by the incorporation of the DNA into the procapsid. Since the *att*B-Ad infectious cycle is delayed without mutating any viral proteins of the packaging complex, these vectors could also be used to study basic aspects of adenovirus assembly.

The protein content of *att*B-Ad particles isolated at 36 and 56 hpi was examined. At 36 hpi *att*B-Ad presented a viral protein pattern similar to Ad5/ts369 at the restrictive temperature indicating an intermediate state of virus maturation. In contrast, the protein pattern of *att*B-Ad at 56 hpi was similar to control Ad5/RFP, though a band likely corresponding to pVI/pVIII precursors was also observed, suggesting that the viral life cycle of *att*B-Ad must be slightly longer than 56 hours. Detailed analysis of two key maturation proteins at 36 hpi showed low amounts of L1-52/55K (either cleaved or uncleaved), and that only ∼50% of pVII protein was cleaved, consistent with the formation of only a few *att*B-helper Ad capsids. However, at 56 hpi, almost all pVII protein was cleaved, while L1-52/55K levels in *att*B-Ad particles were much higher than at 36 hpi. Furthermore, an important proportion of L1-52/55K was still uncleaved, confirming once more that *att*B-Ad had not completed the maturation process at 56 hpi, and that this process may take closer to 60 hours, as observed from the analysis of their life cycle. Collectively, these results demonstrate that the insertion of an *att*B site between the Ad5 ITR and ψ hinders viral DNA packaging and consequently the virion maturation process.

This finding could reflect the altered spacing between ψ and the ITR, but there is considerable flexibility allowed in the location of the packaging signal relative to the genomic terminus, as demonstrated by Hearing and al. [18], so a spacing effect appears unlikely. Rather, results from the EMSA experiment suggest a mechanism by which the *att*B sequence interferes with virus assembly and could be related to the binding of a nuclear protein to this sequence. Due to the proximity of *att*B site to ψ, efficient interaction between packaging complex proteins and ψ would be affected by the binding of the unknown nuclear factor to *att*B. Indeed further analyses are required to identify the unknown nuclear factor.

In conclusion, we have demonstrated that the severe delay in the viral life cycle of *att*B-Ad helper virus is not caused by abnormal viral DNA replication nor by altered levels or availability of viral proteins. However, the packaging process is clearly impaired and it affects the timing of subsequent processes such as maturation of the Ad particles. Thus, at 36 hpi we observed protein aggregates and few unassembled capsids instead of fully matured particles. Only when production times were prolonged to 56 hours was it possible to detect a high percentage of mature and infectious *att*B-Ad virions. Therefore, these results may have important implications for HD-Ad vector technology since this is a novel method based on preferential packaging to produce infectious HD-Ad particles in the absence of helper Ad [10]. Most importantly, it avoids the current limitations associated with standard methods based on recombination-mediated excision of the packaging signal, since it does not require physical removal of contaminating helper virions by ultracentrifugation, and allows the use of scalable downstream methods, such as HPLC purification. Finally, the impairment on genome packaging and capsid maturation processes also makes attB-Ad useful vectors for future studies to better understand the adenovirus life cycle.

## Supporting Information

Figure S1 Western blot analysis of adenoviral protein pVII from CsCl gradients. Ad5/RFP and Ad5/ts369 were harvested at 36 hpi. Ad5/FC31.2 was harvested at 56 hpi. Samples from Ad5/FC31.2 are the intermediate steps of maturation observed in figure 6B.
(TIF)
