# Supplementary figures and images for: Altering the Ad5 Packaging Domain Affects the Maturation of the Ad Particles
Source: PLoS One. 2011 May 18;6(5):e19564. doi: 10.1371/journal.pone.0019564 (PMC3097180; doi:10.1371/journal.pone.0019564)

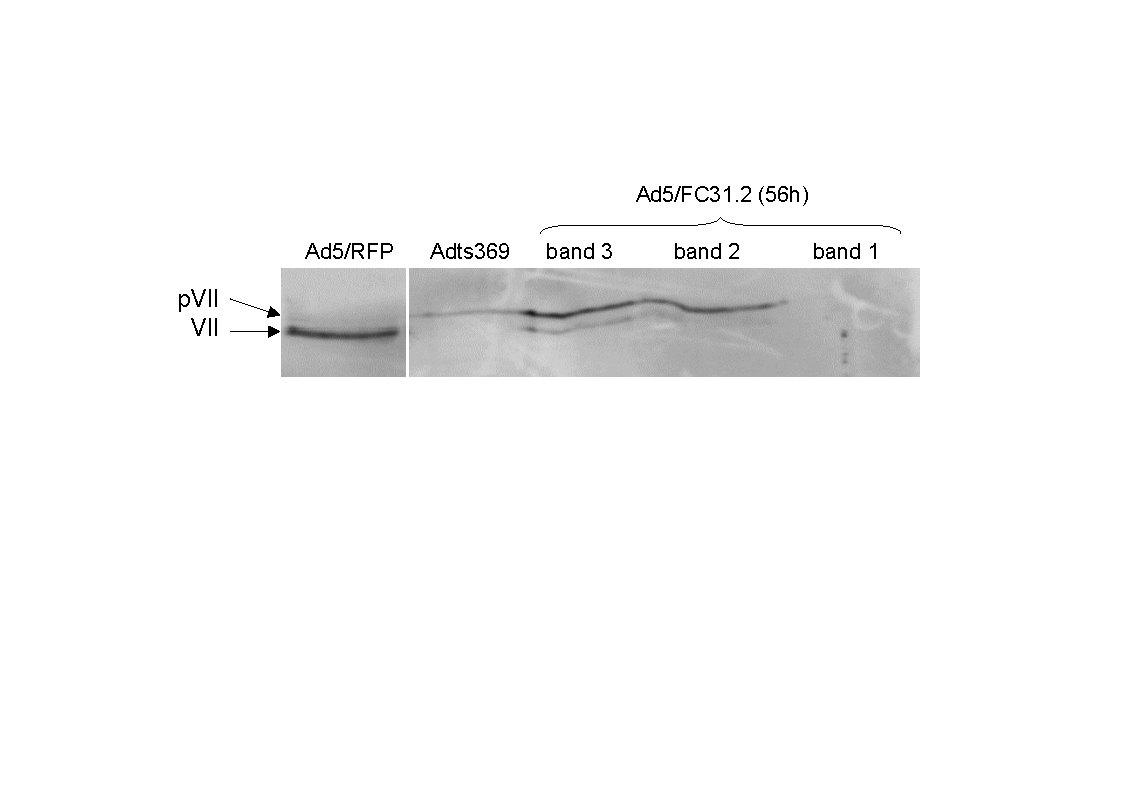

Supplement: Figure S1 — Western blot analysis of adenoviral protein pVII from CsCl gradients. Ad5/RFP and Ad5/ts369 were harvested at 36 hpi. Ad5/FC31.2 was harvested at 56 hpi. Samples from Ad5/FC31.2 are the intermediate steps of maturation observed in figure 6B. (TIF) [file pone.0019564.s001.tif]
